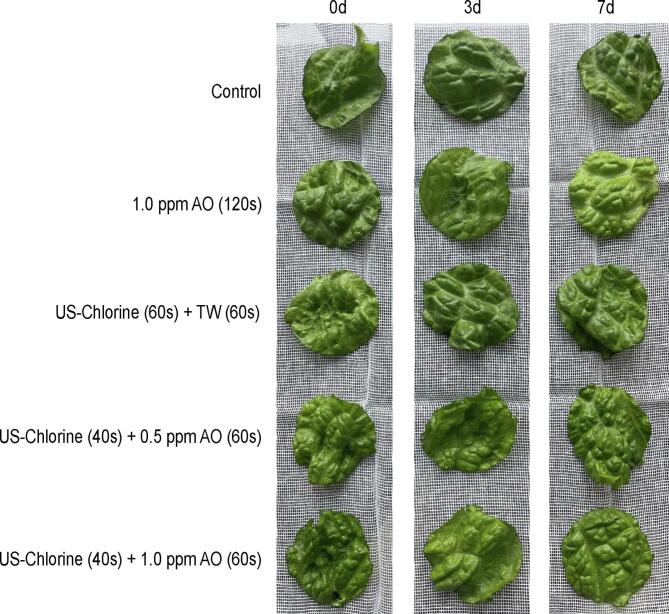# Use of aqueous ozone rinsing to improve the disinfection efficacy and shorten the processing time of ultrasound-assisted washing of fresh produce

**DOI:** 10.1016/j.ultsonch.2022.105931

**Published:** 2022-01-23

**Authors:** Yeting Sun, Zhaoxia Wu, Yangyang Zhang, Jiayi Wang

**Affiliations:** a College of Food and Chemical Engineering, Shaoyang University, Shaoyang 422000, China; b College of Food Science, Shenyang Agricultural University, Shenyang 110000, China; c Beijing Vegetable Research Center, Beijing Academy of Agriculture and Forestry Sciences, Beijing 100097, China

**Keywords:** Ultrasonication, Free chlorine, Disinfection, Aqueous ozone, Fresh-cut

## Abstract

In minimal processing industry, chlorine is widely used in the disinfection process and ultrasound (US) increases the disinfection efficacy of chlorine and reduces the cross-contamination incidence during washing. Tap water (TW), which has no disinfection effect, is generally used to rinse off sanitizer residues on the surface of disinfected fresh-cut vegetables. In this study, aqueous ozone (AO), a low-cost and residue-free sanitizer, was used to replace TW rinsing in combination with US (28 kHz)–chlorine (free chlorine [FC] at 10 ppm, a concentration recommended for industrial use) for the disinfection of fresh-cut lettuce as a model. US–chlorine (40 s) + 2.0 ppm AO (60 s) treatment resulted in browning spots on lettuce surface at the end of storage. In contrast, US–chlorine (40 s) + 1.0 ppm AO (60 s) did not lead to deterioration of the sensory quality (sensory crispness, color, and flavor) and a change in total color difference, and the activities of browning-related enzymes were significantly lower. Moreover, US–chlorine (40 s) + 1.0 ppm of AO (60 s) treatment led to significantly lower counts of *Escherichia coli* O157:H7, *Salmonella* Typhimurium, aerobic mesophilic (AMC), and molds and yeasts (M&Y) on days 0–7 than US–chlorine (60 s) + TW (60 s) and single 1.0 ppm AO (120 s) treatments, suggesting that AO provided an additional disinfection effect over TW, while reducing the overall processing time by 20 s. Cell membrane permeability analysis (alkaline phosphatase, protein, nucleotide, and adenosine triphosphate leakage) showed that the combination with 1.0 ppm AO caused more severe cell membrane damage in *E. coli* O157:H7 and *S*. Typhimurium, explaining the higher disinfection efficacy. 16S rRNA sequencing revealed that following US–chlorine (40 s) + 1.0 ppm of AO (60 s) treatment, *Massilia* and *Acinetobacter* had higher relative abundances (RAs) on day 7 than after US–chlorine (60 s) + TW (60 s) treatment, whereas the RAs of *Escherichia–Shigella* was significantly lower, indicating that the former treatment has a superior capacity in maintaining a stable microbial composition. This explains from an ecological point of view why US–chlorine (40 s) + 1.0 ppm of AO (60 s) led to the lowest AMC and M&Y counts during storage. The study results provide evidence that AO has potential as an alternative to TW rinsing to increase the disinfection efficacy of US–chlorine.

## Highlights

• AO rinsing is an alternative to TW rinsing.
• Ultrasonication (US)-chlorine plus aqueous ozone (AO) showed better disinfection efficacy than the other treatments evaluated.
• US-chlorine plus AO had no negative effects on the quality in green leaf lettuce.

## 1 Introduction

Fresh produce are important sources of vitamins, minerals, and fibers [1]. Compared with thermally processed produce, fresh produce possess higher nutritional values. Fresh-cut produce are widely consumed in Europe and the USA owing to their variety and convenience. In recent years, the demand for fresh-cut produce is increasing in developing countries, including the first-tier cities of Shanghai, Beijing, Guangzhou, and Shenzhen in China. However, food safety is a major problem with fresh-cut produce because of the risk of foodborne pathogen contamination [2]. *Salmonella* and *Escherichia coli* O157:H7 are the most common foodborne pathogens. According to statistics, 39.5 % and 42.6 % of foodborne illness outbreaks in the USA and Europe were caused by the consumption of fresh produce, and 30.87 % and 47.65 % of cases in the USA and 8.33 % and 47.62 % of cases in Europe were caused by *E. coli* and *Salmonella*, respectively [3]. In Rwanda, 15 % of agricultural products were contaminated with pathogens, with *E. coli* accounting for the largest share at 6.1 % [4].

There are many emerging technologies for the disinfection of fresh-cut produce, including biological techniques, such as phages and microbial–microbial interaction, and physical techniques, including pulsed light and cold plasma [[5], [6]]. However, chemical disinfection techniques are the most widely used in practice because of their low cost and easy availability [[7], [8]]. In the minimal processing industry, washing water is recirculated for re-use and thus, when infected vegetables are washed in the washing tank, the pathogens present on their surface can be transmitted via the washing water, leading to cross-contamination. Hence, immediate pathogen inactivation in the washing water is a key concern [[8], [9]]. Moreover, even at high concentrations, sanitizers in the washing tank fail to inactivate pathogens to an undetectable level [10]. Residual pathogens are not permitted by all national standards, and the high proliferation rate of pathogens can cause a rebound on fresh produce during storage [8]. Furthermore, the industry aims to reduce disinfectant dosages to reduce the costs and suppress by-product formation [[11], [12]]. Therefore, studies have focused on using minimal doses of chemical disinfectants, often in combination with other technologies, to reduce the cross-contamination incidence [[13], [14]]. Among the chemical disinfectants available, chlorine compounds are the most effective and least costly [15]. Sodium hypochlorite is the most widely used chlorine disinfectant, and several studies have shown that a free chlorine (FC) concentration around 10 ppm can prevent cross-contamination [[7], [12], [15]]. Ultrasound (US) technology has been widely used for the disinfection of fresh produce. When combined with FC, US can effectively prevent cross-contamination and improve the disinfection efficacy for fresh produce [[16], [17]]. Hence, US is considered a low-cost and effective auxiliary technology for fresh produce disinfection.

**Fig. 1:**
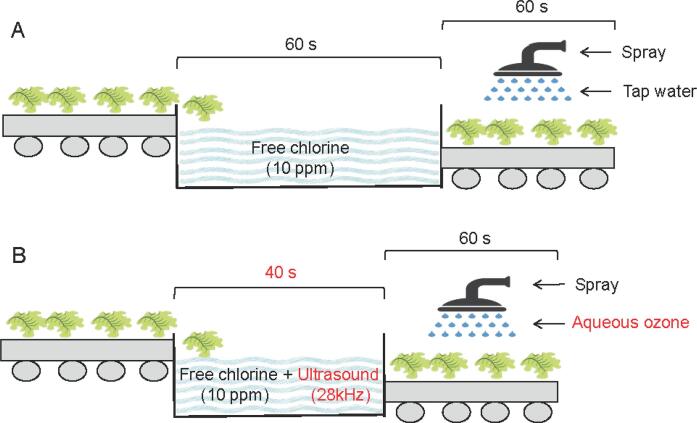
Schematic diagram of the fresh-cut vegetable process proposed in this study in comparison with the existing fresh-cut vegetable process. (A) Existing process combining free chlorine washing with tap water rinsing. (B) Proposed process combining ultrasonication–chlorine with aqueous ozone rinsing. The improvements suggested in this study are highlighted in red font.

After fresh-cut vegetables are disinfected in a washing tank, they are rinsed with tap water (TW) to remove disinfectant residues from the vegetable surface (Fig. 1A); however, TW has no disinfection effect [11]. Ozone can be produced from air at low cost and can be dissolved in water. Given its unstable state, aqueous ozone (AO) left on vegetable leaves decomposes into oxygen, making it a residue-free disinfectant [2]. AO has been reported to be effective in controlling the browning of fresh produce [[18], [19]].

In this study, we aimed to evaluate the disinfection efficacy of AO in combination with US–chlorine (Fig. 1B) in fresh-cut lettuce and to evaluate its effects on vegetable quality and the activity of browning-related enzymes (peroxidase (POD) and polyphenol oxidase (PPO)). To gain a deeper understanding of the effects of the combination treatment on the microbial composition on the lettuce leaves, 16S rRNA sequencing was used to analyze microbial compositional changes. Given that the practical implications of enhanced disinfection efficacy and improved quality would have a limited advantage, we also aimed to reduce the processing time in the washing step (Fig. 1B) to explore whether US–chlorine + AO can not only provide enhanced disinfection efficacy, but also increase productivity.

## 2 Materials and methods

### 2.1 Sample preparation

Lettuce is the most widely consumed fresh-cut vegetable, and green leaf lettuce has the thinnest leaves and most fragile texture among various lettuce varieties; therefore, we used lettuce as a model in this study as it allows for setting stringent standards for quality analysis. Green leaf lettuce (*Lactuca sativa* var. *Crispa* L.) was purchased from a local market on the day of the experiment. After removing the stem, damaged external leaves, and internal baby leaves, the remaining leaves were rinsed under TW for 30 s to remove the dirt and cut using a 3.0-cm-diameter circular cutting edge. The cut leaves were dewatered in a sterilized (sprayed with 75 % ethanol) salad manual spinner for use in the experiments [[2], [20]].

### 2.2 Pathogen inoculation

Pathogen inoculation was carried out according to our previous reports [[21], [22]]. *E. coli* O157:H7 (NCTC12900) and *Salmonella* Typhimurium (ATCC14028) were purified on modified sorbitol MacConkey agar and xylose lysine deoxycholate agar, respectively, and after culture at 37 °C for 24 h, one colony was inoculated into tryptic soy broth and cultured under shaking at 120 rpm at 37 °C for 8 h. Then, 5 mL of diluted bacterial suspension (∼10^9^ CFU/mL) was mixed with 200 mL of 0.85 % NaCl in a sterile stomacher bag. Subsequently, 10 g of lettuce sample was added and manually massaged for 10 min. The sample was then placed in a sterile plastic box and stored in a biosafety cabinet for air drying. Thereafter, the sample was stored at 4 °C for 24 h to allow sufficient microbial attachment, and the final microbial count on sample was 10^6^–10^7^ CFU/g.

### 2.3 Disinfection experiment

In this experiment, the concentration of FC (10 ppm), washing time (60 s), and TW rinsing time (60 s) were based on parameters reported for practical application [[7], [11], [15], [23]].

#### 2.3.1 Washing water preparation

The washing water used for fresh-cut vegetables is recycled and the soluble matter from the cut edge can lead to a high chemical oxygen demand (COD) in the washing water, consuming oxidizing sanitizer; therefore, fresh produce homogenates have been used in many experimental studies to prepare washing water, rather than clean water [[15], [24], [25]]. In this study, washing water with two COD concentrations was prepared based on COD scenarios reported in practical applications to assess the disinfection efficacy under different COD scenarios [[15], [25]]. Lettuce leaves were prepared as described in Section 2.1. Thereafter, they were immediately placed in an analytical mill (A11 basic; IKA, Germany) and processed for 20 s. The resulting lettuce homogenate was filtered under vacuum and sterilized for 15 min by subjecting it to a temperature of 121 °C. Finally, it was stored at −20 °C until use. Before the experiment, sterilized TW was mixed with the homogenate and the COD was adjusted to 703 ± 16 and 1504 ± 11 mg/L. The concentration of FC and pH in the washing water was adjusted to 13.87 ± 1.21 mg/L, and 5.5 using sodium hypochlorite (Sinopharm, Beijing, China) and phosphoric acid, respectively [11]. The COD and FC concentrations were determined using a COD and *N,N*-diethyl-p-phenylenediamine test kit (Lohand, Hangzhou, China), respectively.

#### 2.3.2 Disinfection

A 100-g lettuce sample was placed in a stainless steel cage (25 × 15 × 5 cm) that was placed in an ultrasonic washer (JM-30D-28; Skymen, Shenzhen, China) containing 8 L of washing water. Then, a submersible pump (3,500 L/h; Chuangning, China) was placed at the bottom of the washer to generate water flow. US treatment was conducted for 60 s or 40 s at a optimized condition (28 kHz, 300 W).

TW and AO rinsing steps were performed using a spraying system (Sushen, Zhejiang, China) consisting of a self-priming pump, a spray nozzle, and a water bucket (containing 10 L of TW). The ozone generator (10 g/h; Shenghuan, Guangzhou, China) was connected to a sparger (Juren, China) to dissolve the ozone in the water to prepare 0.5, 1, and 2 mg/L AO. The AO concentration was determined according to a report by Barder et al. [26]. Following the US–chlorine treatment, the sample was placed on a shaker (Jintan, Changzhou, China) at 120 rpm, the spray nozzle was fixed at 70 cm above the sample, and the self-priming pump was placed in the bucket to initiate rinsing at a rate of 0.75 L/min. After rinsing for 1 min, the sample was dewatered in a sterilized manual salad spinner. Then, the sample was placed in polyethylene terephthalate plastic box (18 × 13 × 4 cm) (30 g per box), sealed using polyvinyl chloride plastic film, and stored at 4 °C [2].

#### 2.3.3 Microbial count analysis

Microbiological analysis was performed on days 0, 3, and 7. A sample (15 g) was placed in a stomacher bag containing 135 mL of 0.85 % NaCl and homogenized for 2 min, followed by serial dilution. Then, 0.1 mL of the diluted bacterial suspension was surface-plated on modified sorbitol MacConkey agar (Hopebio, Qingdao, China) or xylose lysine deoxycholate agar (Hopebio) and incubated at 37 °C for 24 h to count *E. coli* O157:H7 and *Salmonella* Typhimurium, respectively. For naturally present microbes, 0.1 mL of suspension was surface-plated on Rose Bengal agar (Hopebio) and incubated at 30 °C for 3 days to quantify molds and yeasts (M&Y). In addition, 1 mL of the suspension was pour-plated onto plate count agar (Hopebio) and incubated at 37 °C for 2 days to obtain the aerobic mesophilic count (AMC). All results are expressed as log CFU/g.

### 2.4 Sensory evaluation and total color difference (TCD) analysis

Ten samples were randomly selected from each package, and the color values of L*, a*, and b* were determined using a colorimeter (CR400; Konica Minolta, Japan). The TCD was calculated using the following formula:

$$TCD=\sqrt{{\left(L_{1}^{*}-L_{0}^{*}\right)}^{2}+{\left(a_{1}^{*}-a_{0}^{*}\right)}^{2}+{\left(b_{1}^{*}-b_{0}^{*}\right)}^{2}}$$

where $L_{0}^{*}$, $a_{0}^{*}$, and $b_{0}^{*}$ denote Hunter’s color values corresponding to a reference, and $a_{1}^{*}$, and $b_{1}^{*}$ denote Hunter’s color values corresponding to the sample on the sampling day.

Sensory evaluation was carried out in a room with white walls and no windows and under illumination by a 40-W fluorescent lamp following the method described by Allende et al. [27], with some modifications. Eight trained sensory panelists scored the sensory color, crispness, and flavor, where a score of “0″ indicated very poor quality and poor product characteristics, “5” was the acceptability threshold, and “10” indicated very good quality with good product characteristics. Before scoring, the bottom of the white plates containing the samples were numbered and reordered. During scoring, only one person was allowed into the room and was asked to avoid communication with the other panelists after evaluation. For flavor analysis, the panelists gargled three times using drinking water after each evaluation, and the next sample was evaluated after a 30-s interval.

### 2.5 PPO and POD analyses

PPO and POD were analyzed according to the method reported by Chen et al. [28], with minor modifications. Lettuce samples were grounded in liquid nitrogen, and the ground powder was transferred to precooled centrifuge tubes. Then, 0.2 mol/L of sodium phosphate buffer (containing 0.5 % polyvinylpyrrolidone; pH 7.0) was added at a ratio of 1:2 (w/v) and the sample was centrifuged at 12000 rpm, 4 °C for 20 min.

For PPO analysis, 0.8 mL of the supernatant was mixed with 2.4 mL of substrate solution (0.02 mol/L catechol dissolved in 0.05 mol/L sodium phosphate buffer; pH 7.0), and the absorbance at 420 nm was measured. The blank consisted of 0.8 mL of sodium phosphate buffer. One unit of enzyme activity was defined as an increase in the absorbance of 0.001 per mL of enzyme per minute.

For POD analysis, 0.2 mL of the supernatant was mixed with 2.8 mL of substrate solution (25 mmol/L guaiacol and 25 mmol/L hydrogen peroxide dissolved in 0.05 mol/L sodium phosphate buffer; pH 7.0), and the absorbance at 470 nm was measured. The blank consisted of 0.2 mL of sodium phosphate buffer. One unit of enzyme activity was defined as an increase in the absorbance of 0.01 per mL of enzyme per minute.

### 2.6 Cell membranes integrity analysis

Cell membrane integrity was analyzed according to the method reported by Wang et al. [29]. Sterilized TW (120 s) alone was included as a control treatment, and 1 ppm AO (120 s), US–chlorine (60 s) + TW (60 s), and US–chlorine (40 s) + 1 ppm AO (60 s) were the experimental treatments. Bacterial suspensions of *E. coli* O157:H7 and *S.* Typhimurium were prepared as described in section 2.2. Bacterial pellets were washed three times with 0.85 % NaCl, centrifuged at 12,000 × *g*, 4 °C for 10 min, and resuspended in a centrifuge tube using sterile distilled water. After adjusting the FC in the tube to 10 ppm, the tube was placed immediately in the center of the ultrasonic washer (containing 8 L washing water) set at 28 kHz and 300 W. After US–chlorine treatment, prepared AO was added to the tube at a final concentration of 1 ppm. After AO treatment, 5 g/L sodium thiosulphate was added at a ratio of 1:2 (v/v) to neutralize the disinfectant, and the sample was filtered through a 0.22-µm sterilized membrane (HKM, Guangzhou, China). Protein and nucleotide concentrations in the supernatant were determined by the microprotein kit (Jiancheng, Nanjing, China) and by measuring the absorbance at 260 nm, respectively. Adenosine triphosphate (ATP) and alkaline phosphatase (AKP) were determined using test kits (Jiancheng).

### 2.7 Microbial composition analysis

#### 2.7.1 DNA extraction

US–chlorine treatment was performed under high COD (1500 mg/L) in all treatment groups. Thirty milliliters of the bacterial suspension prepared as described in section 2.3.3 was filtered through sterilized 0.22-µm membranes (HKM), using a fresh filter membrane after every 15 mL of liquid filtered. Subsequently, the membranes were placed at –80 °C until use. DNA was extracted from the bacteria present on the membranes using a rapid DNA extraction kit (MP Biomedicals, Santa Ana, CA, USA) according to the manufacturer’s instructions.

#### 2.7.2 Amplicon pyrosequencing

The 16S rRNA gene V3–V4 region was amplified by PCR using the 338F and 806R. Seven-base pair barcodes were incorporated into the primers for multiplex sequencing. The PCR mixture contained 1 μL of forward primer (10 μM), 1 μL of reverse primer (10 μM), 1 2 μL of DNA template, 0.25 μL of Q5 high-fidelity DNA polymerase (5 U/μL^–^), 5 μL of Q5 reaction buffer (5 × ), 5 μL of Q5 high-fidelity GC buffer (5 × ), 2 μL of deoxynucleoside triphosphate (2.5 mM), and 8.75 mL of double-distilled water. The thermal cycling conditions were as follows: initial denaturation at 98 °C for 2 min followed by 25 cycles of denaturation at 98 °C for 15 s, annealing at 55 °C for 30 s, and extension at 72 °C for 30 s, and a final extension at 72 °C for 5 min. The PCR amplicons were purified using Agencourt AMPure Beads (Beckman Coulter, Indianapolis, IN, USA) and quantified using a PicoGreen dsDNA Assay Kit (Invitrogen, Carlsbad, CA, USA). After pooling in equal amounts, the amplicons were sequenced on an Illumina MiSeq platform.

#### 2.7.3 Sequence analysis

The QIIME v.1.8.0 pipeline was employed to control the quality of sequencing data, as reported by Caporaso et al. [30]. In brief, reads with exact matches were classified as high-quality sequences, and reads shorter than 150 bp, average Phred score < 20, and containing ambiguous bases and mononucleotide repeats (>8 bp) were classified as low-quality sequences. After fast length adjustment of short reads [31] and pair-end reads, and chimera detection, the sequences were clustered as operational taxonomic units (OTUs) at 97 % sequence identity using UCLUST [32]. Then, default parameters were used to select a representative sequence, which was used to classify the OTU against the Greengenes database based on the best BLAST hit. OTUs containing < 0.0001 % of the total sequences were removed. The sequencing depth difference across the samples was minimized by resampling 100 OTU subsets under a 90 % sequencing depth. ACE and Chao1 index values were calculated using QIIME. Spearman’s rank correlation (|rho| > 0.6; *P* < 0.01) was analyzed using Mothur.

### 2.8 Statistical analysis

All experiments, except 16S rRNA sequencing, were replicated three times independently, and 16S rRNA sequencing was replicated six times independently. TW treatment was included as a control. All data are expressed as mean ± standard deviation. Group means were compared using Duncan’s multiple range test. Significant differences between days 0 and 7 were evaluated using the independent-samples *t*-test. *P* < 0.05 was considered significant. Statistical analyses were conducted using SPSS 22 (SPSS, Chicago, IL, USA).

## 3 Results and discussion

### 3.1 Disinfection efficacy of US plus chlorine

**Fig. 2:**
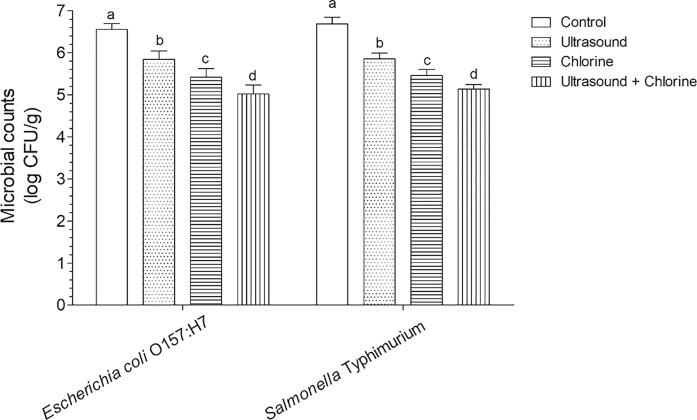
Effects of US, chlorine, and US–chlorine treatments on pathogen abundance on the surface of fresh-cut lettuce. Different lowercase letters within the same group indicate significant differences (*P* < 0.05).

Before use in combination with AO, we clarified whether the US–chlorine treatment had a better disinfection effect than the US- or chlorine-only treatments. Thus, a 60-s treatment time was selected. The results showed that chlorine or US treatment reduced *E. coli* O157:H7 and *S.* Typhimurium by <1 log CFU/g. When the two treatments were combined, the disinfection effect was significantly enhanced (Fig. 2), which was consistent with results reported by Huang et al. [17]. Therefore, US–chlorine was used in subsequent experiments.

### 3.2 Effects of the treatments on the quality of fresh-cut lettuce

#### 3.2.1 Effects on color and sensory quality

**Fig. 3:**
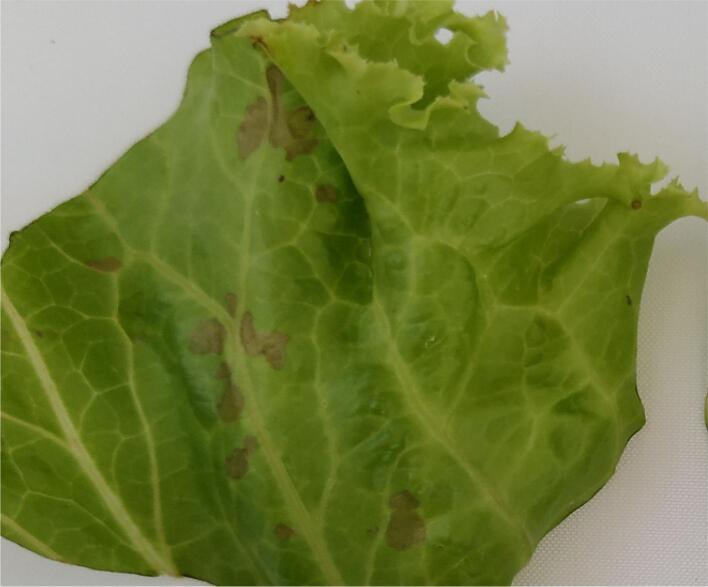
Browning spots on fresh-cut lettuce after treatment with ultrasonication–chlorine (1500 mg/L chemical oxygen demand) plus 2 ppm aqueous ozone.

**Fig. 4:**
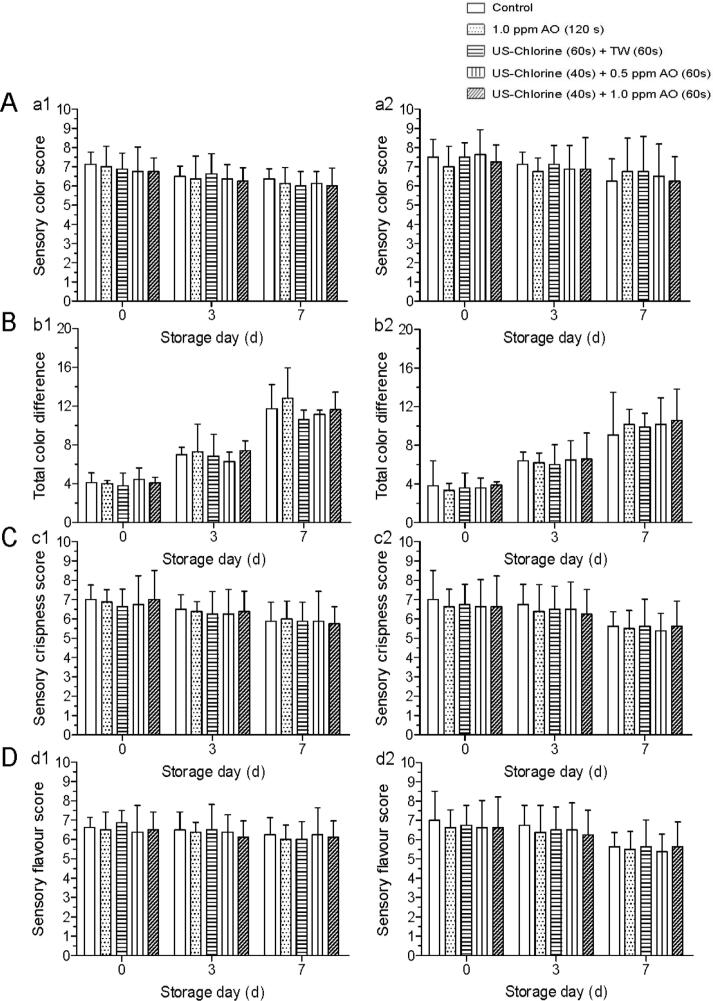
Effects of indicated treatments on sensory quality and total color difference of fresh-cut lettuce during storage (0–7 d). (A) Sensory color, (B) Total color difference, (C) Sensory crispness, (D) Sensory flavor. Figures a1, b1, c1, and d1 indicate the parameter detected under COD condition of 700 mg/L; a2, b2, c2, and d2 indicate the parameter detected under COD condition of 1500 mg/L. No significant differences were observed between the groups (*P* > 0.05) on the same day. US, ultrasonication; TW, tap water; AO, aqueous ozone; COD, chemical oxygen demand.

Sensory quality is the major factor determining a consumer’s decision to purchase. According to previous reports, US treatment resulted in significantly lower sensory scores for Romaine lettuce than Iceberg lettuce, mainly because Romaine lettuce leaves are thinner than those of Iceberg lettuce and therefore more susceptible to damage [33]. As a strong oxidizing agent, AO can cause produce quality deterioration when used at high concentrations. Martínez-Sánchez et al. [34] found that rocket salad treated with 10 ppm AO had lower ascorbic acid contents during storage than salad treated with other sanitizers (Purac, Sanova, Tsunami, and sodium hypochlorite). Moreover, 10 ppm AO caused slight damage to table grapes [35]. In the present study, when US–chlorine was combined with 2 ppm AO, browning spots were observed on the lettuce leaf surfaces on day 7 (Fig. 3); this was contrary to the observations made for lettuce leaves following other treatments between days 0 and 7 (Figure S1). In a previous study, FC did not negatively affect lettuce sensory quality at concentrations up to 200 ppm [36], whereas only 10 ppm FC was used in this study. Hence, the browning phenomenon was likely caused by US and AO. We selected 0.5 and 1 ppm AO for subsequent experiments, and the results obtained showed no significant differences between the US–chlorine + AO- and control-treated fresh-cut lettuce samples with respect to sensory color, crispness, flavor scores, and TCD during storage (0–7 d) (Fig. 4A–D). Additionally, quality loss was not observed after rinsing with 1 ppm AO for 120 s; this is consistent with the findings reported by Baur et al. [36].

#### 3.2.2 Effects of the treatments on browning-related enzyme activities

**Fig. 5:**
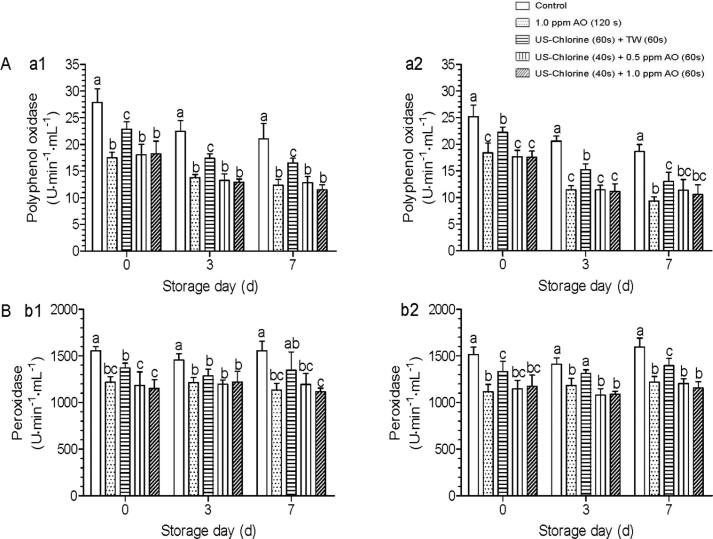
Effects of indicated treatments on peroxidase and polyphenol oxidase activities in fresh-cut lettuce during storage (0–7 d). The different lowercase letters within the same group indicate significant differences (*P* < 0.05). (A) Polyphenol oxidase activity, (B) Peroxidase activity. Figures a1 and b1 represent parameters detected under COD condition of 700 mg/L; a2 and b2 represent parameters detected under COD condition of 1500 mg/L. US, ultrasonication; TW, tap water; AO, aqueous ozone; COD, chemical oxygen demand.

In uncut produce, PPO and POD do not come in contact with oxygen; thus, browning does not occur; however, after cutting, these react with oxygen to form quinones, which bring about color and flavor deterioration [37]. The shear force induced by low frequency US can inactivate PPO and POD at the cutting edge of fresh produce [38]. When US treatment was combined with other treatment methods, the anti-browning effects could be improved. For example, natural product *Sonchus oleraceus* L. and propolis extract can improved the inactivation activity of US against browning-related enzymes (PPO and POD) in fresh-cut potatoes and mixed salads, respectively [[39], [40]]. Further, food additives, such as ascorbic acid and cystine can improve the anti-browning effects of US during the treatment of fresh-cut apples and lotus root, respectively [[41], [42]]. It is also known that oxidizing disinfectants, such as chlorine dioxide and AO inhibit fresh-cut lettuce browning [28]; however, there are few studies on the combined use of such oxidizing agents with US to inhibit lettuce browning. In this study, we combined US with chlorine to wash fresh-cut lettuce and further rinsed it with AO. In this regard, browning-related enzyme analysis showed that the US–Chlorine + TW treatment can significantly inhibit the activity of PPO and POD between days 0 and 7 (except POD at d7 under the condition of 700 mg/L COD; Fig. 5b1). When the US–Chlorine treatment was combined with AO, inhibitory activity against PPO and POD was significantly improved compared with that corresponding to the US–Chlorine + TW treatment, on day 0. Further, during storage (days 3–7), the enzyme activity corresponding to this combined treatment was still significantly lower than that corresponding to the control, suggesting that rinsing with AO can reduce the incidence of browning.

The cut area of fresh-cut lettuce exposed to US is smaller than those of fresh-cut potatoes or fresh-cut apples. Meanwhile, lettuce surface damage caused by US should be considered given that browning can still occur on lettuce surface after such US-induced damaging [33]; thus, moderate disinfection treatment is important to inhibit browning without destroying the quality of the lettuce. In this study, we observed that the US–Chlorine + TW treatment did not negatively affect the sensory quality and TCD of fresh-cut lettuce, and after further rinsing with AO, these parameters were not further affected. The PPO and POD activities corresponding to the fresh-cut lettuce samples treated with US–Chlorine + 0.5 ppm AO, US–Chlorine + 1.0 ppm AO, and AO-only showed no significant differences regardless of the different COD values. This observation could be primarily attributed to is the fact that after washing with US–Chlorine, most of the organic matter accumulated on lettuce surface, while a limited amount accumulated on cutting edge, and during the rinsing step, the small-area cutting edge only consumed little AO; thus, similar PPO and POD activity was observed after rinsing with 1 or 0.5 ppm AO.

### 3.3 Disinfection efficacy of the treatments

**Fig. 6:**
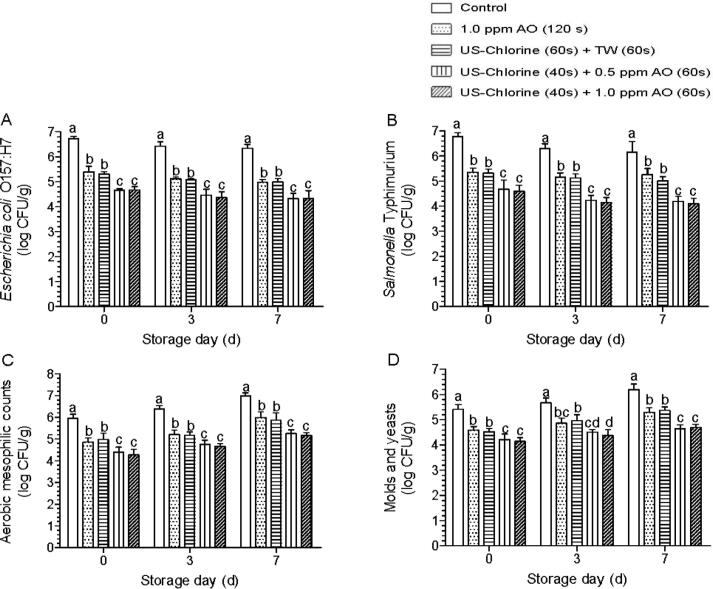
Effects of the indicated treatments on inoculated pathogens and naturally present microbes on fresh-cut lettuce leaves under low chemical oxygen demand (700 mg/L) condition. (A) *Escherichia coli* O157:H7, (B) *Salmonella* Typhimurium, (C) aerobic mesophilic counts, (D) molds and yeasts. Different lowercase letters within the same group indicate significant differences (*P* < 0.05). US, ultrasonication; TW, tap water; AO: aqueous ozone.

Hurdle technology often does not provide synergistic disinfection effects, but can provide additional microbial suppression when compared with single treatments [43]. The results showed that US–chlorine + TW reduced *E. coli* O157:H7 and *S.* Typhimurium by 1.41 and 1.46 log CFU/g, respectively, on day 0 (Fig. 6A, B), with a similar disinfection effect as that of single AO treatment under low COD condition. During storage, the counts of these two pathogens were not significantly different between these two treatments. When AO was used to replace TW rinsing, the disinfection effect against the two pathogens was significantly improved. On day 0, the reduction exceeded 2 log CFU/g for both pathogens, and during storage, the microbial counts in the US–chlorine + AO group were significantly lower than those in the AO and US–chlorine + TW group. However, the microbial counts for both pathogens did not significantly differ between US–chlorine + 0.5 ppm AO and US–chlorine + 1.0 ppm AO on days 0–7. For AMC and M&Y, the counts were significantly lower after US–chlorine + AO treatment than after AO and US–chlorine + TW treatment on day 0 and during storage (Fig. 6C, D). However, US–chlorine + 0.5 ppm AO had similar disinfection effects against AMC and M&Y as US–chlorine + 1.0 ppm AO.

**Fig. 7:**
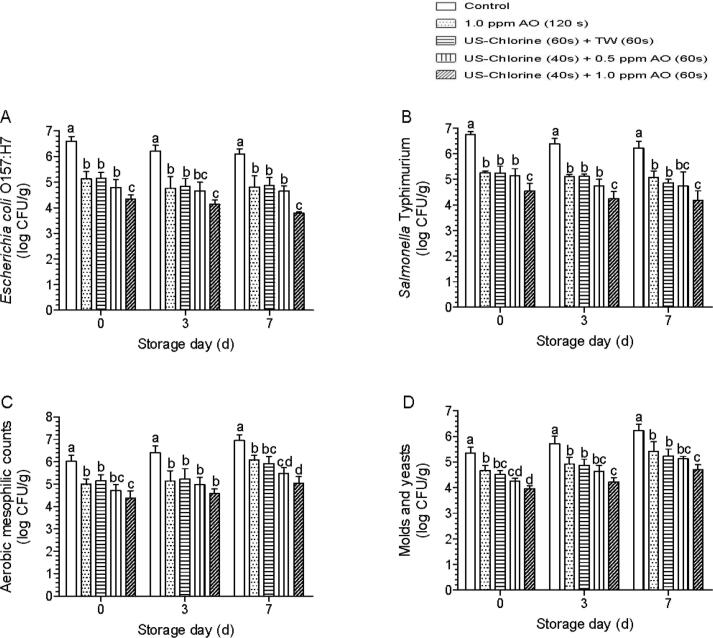
Effects of the indicated treatments on the inactivation of inoculated pathogens and naturally present microbes on the surface of fresh-cut lettuce under a high chemical oxygen demand concentration (1500 mg/L) in the washing water. (A) *Escherichia coli* O157:H7, (B) *Salmonella* Typhimurium, (C) aerobic mesophilic counts, (D) molds and yeasts. Different lowercase letters within the same group indicate significant differences (*P* < 0.05). US, ultrasonication; TW, tap water; AO: aqueous ozone.

When the washing water had a high COD, the US–chlorine + TW treatment reduced *E. coli* O157:H7, *S.* Typhimurium, AMC, and M&Y by 1.45, 1.50, 0.87, and 0.82 log CFU/g, respectively, which is consistent with the microbial reduction caused by single AO rinsing, and until the end of storage, there was no significant difference between these two treatments (Fig. 7). When AO was combined with US–chlorine, the counts for AMC and M&Y were significantly lower than those after the US–chlorine + TW and AO treatments only when the AO concentration was 1.0 ppm (Fig. 7). These results were inconsistent with those in Fig. 6.

Ozone is sensitive to organic matter and reacts not only with microorganisms, but also with dirt and pesticide residues present on the produce surface. Our previous study showed that 2 ppm AO is effective in degrading malathion and carbosulfan on pak choi, but has a limited effect in disinfecting AMC [44]. Numerous studies have reported that, even with high AO concentrations and long treatment times, the disinfection effect of AO is limited. Washing peppers with 3.5 ppm AO for 30 min insignificantly reduced the microbial load [45]. AO at 20 ppm reduced M&Y on strawberry surfaces by only 0.78 log CFU/g, and 10 ppm AO reduced AMC on lettuce by only 0.99 log CFU/g [46]. In durum wheat, washing with 16.5 ppm AO reduced M&Y by only 0.5 log CFU/g [47]. In the current study, after disinfection in the low COD washing water under the US–chlorine treatment, little organic matter was left on the lettuce surfaces and thus in the next step, a limited part of the AO was consumed by the organic matter. However, when the COD concentration of the washing water was increased, a larger part of the AO was consumed by the organic matter present on the lettuce surfaces and 0.5 ppm AO was ineffective for disinfection. This explains why the disinfection effect of US–chlorine + 1.0 ppm AO was significantly higher than that of US–chlorine + 0.5 ppm AO on day 0 under high COD condition, whereas under low COD condition, no significant difference was observed.

The solubility of ozone is affected by water purity, pH, and temperature. Many studies have used distilled water as the dissolving medium for ozone. However, in practical applications, the use of TW is widely accepted, and therefore, TW was used in this study. Ozone is typically dissolved in water using a venturi, sparger, and a gas–liquid mixing pump. Although it is possible to prepare AO at a high concentration (>3 ppm) on a small scale when using this equipment, this approach is limited in use on a large scale. According to our previous experiments (unpublished data), when using a most efficient gas–liquid mixing pump (a high-speed rotating turbine in the pump to produce negative pressure, which effectively mixes the gas and liquid in the mixing chamber), the AO concentration in 40 L of TW only reached 2 ppm after 15 min of circulation and did not increase after longer circulation. Therefore, the use of AO < 3 ppm meets practical application requirements. In this study, the results of the microbiological experiments showed that, regardless of the COD of the washing water under US–chlorine treatment, subsequent rinsing with 1 ppm AO significantly improved the disinfection efficacy against *E. coli* O157:H7, *S.* Typhimurium, AMC, and M&Y compared to rinsing with TW.

### 3.4 Cell membrane damage caused by the treatments

**Fig. 8:**
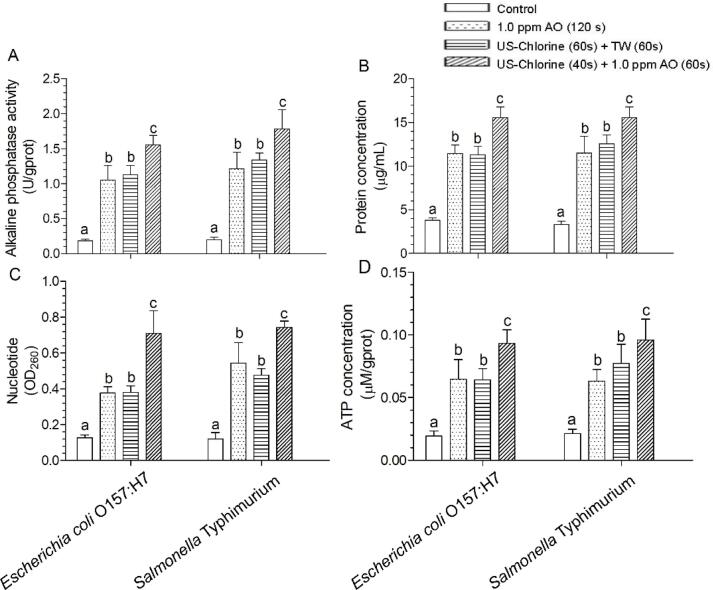
Effects of the indicated treatments on the permeability of *Escherichia coli* O157:H7 and *Salmonella* Typhimurium cell membranes. (A) alkaline phosphatase activity, (B) protein content, (C) nucleotide, (D) adenosine triphosphate. Different lowercase letters within the same group indicate significant differences (*P* < 0.05). US, ultrasonication; TW, tap water; AO: aqueous ozone.

In this study, US and chlorine were combined during the washing step, and the addition of AO further improved disinfection efficacy against *E. coli* O157:H7 and *S.* Typhimurium. Among these combinations, the US–chlorine + 1.0 ppm AO treatment resulted in the lowest counts of *E. coli* O157:H7 and *S.* Typhimurium considering the two COD conditions; thus, the US–chlorine + 1.0 ppm AO treatment was selected for comparison with the AO-only and US–chlorine + TW treatments in terms of their abilities to damage pathogen membranes. When the cell membrane is broken, ATP, AKP, proteins, and nucleotides leak out, and the extent of this leakage reflects the degree of change in the cell membrane integrity [[29], [48]]. Thus, our results indicated significantly higher ATP, AKP, proteins, and nucleotides leakage levels after US–chlorine + 1.0 ppm AO treatment than after the US–chlorine + TW treatment (Fig. 8), indicating that the former caused more serious membrane damage, and compared with the AO-only treatment, the US–chlorine + 1.0 ppm AO treatment also induced more serious membrane damage, even when the treatment duration was <20 s.

The antibacterial mode of action of US includes sonoporation and sonochemistry. When low-frequency (20–100 kHz) ultrasound is used, collapsed cativation bubbles can induce shear force and shock waves that induce membrane perforation; while a high frequency can induce the generation of hydrogen peroxide as well as ROS, which inactivate pathogens; this is known as sonochemistry [[49], [50]]. Further, when using US in combination with other disinfection strategies, low-frequency US is recommended and the cell membrane is considered as main attack target [50]. A recent study reported that US and chlorogenic acid can synergistically inactivate *Staphylococcus aureus*, mainly by disrupting cell membrane permeability [51]. Zhang et al. [52] also reported the synergistic disinfection effects of US and citral against *E. coli* and *Listeria monocytogenes* via the acceleration of cell membrane damage. Furthermore, it has also been reported that in addition to its physical impact on bacterial membrane structures, US can also enhance the dispersion of sanitizer droplets [52]. Additionally, the antibacterial mechanisms of action of chlorine and ozone involve inducing cell membrane oxidation as well as membrane component leakage [1]. In this study, the combination of low frequency US with FC showed that the extent of cell membrane damage caused by the US–Chlorine treatment was similar to that caused by the AO-only treatment even the disinfection time of US–Chlorine treatment was reduced by 60 s. This may be attributed to US simultaneously destroying bacterial cell membrane and enhancing the dispersion of FC, making its contact with the bacterial cell membrane more complete compared with the AO-only treatment. After combining US–Chlorine with AO, AO could further damage cell membrane, leading to the most serious damage even when the overall treatment time was reduced by 20 s.

When fresh produce are contaminated by pathogen, a layer by layer state of the pathogen were formed on produce surface. Use of US and FC alone will detached and inactivate the pathogen from the upper layer, respectively; however, when US and FC are combined, shear force can weaken the adhesion between each pathogen layer, making FC more easy to inactivate the pathogen in deeper layer [50]. After rinsing with 1 ppm AO, AO could further disinfect the pathogen in deeper layer, leading to the highest microbial reduction on lettuce surface. Other studies using a combination of US and an oxidizing chemical sanitizer have also reported enhanced disinfection efficacy for fresh produce. In a previous study, it was observed that the disinfection effects of combinations of US with electrolytic water on *E. coli* in fresh-cut lettuce were stronger than those of the single treatments [53]. Specifically, a combination of US with electrolytic water enhanced disinfection efficacy against *Bacillus cereus* in fresh-cut potatoes to a greater extent than the single treatments [54].

### 3.5 Effects of the treatments on microbial composition

**Fig. 9:**
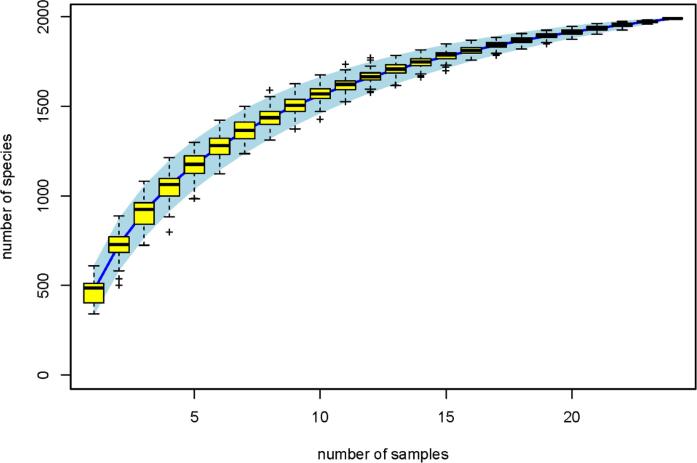
Specaccum species accumulation curve.

The effects of the various treatments on naturally present microbes were analyzed using agar count methods. However, agar methods are limited in analyzing microbial diversity on fresh produce. For example, only 5 % of the fungal species occurring in nature can be cultured on agar [55]. With the development of sequencing technologies, species and composition changes can be deeply investigated. In this study, 16S rRNA sequencing technology was used to analyze the microbial composition between treatment and control groups on days 0–7. A specaccum species accumulation curve (Fig. 9) showed that the numbers of species identified increased with increasing sample number, and when the sample number was 24 (1 control, 3 treatments, 6 replications), the number of species identified reached to 1945, which was consistent with the previous report [20].

The Chao1 and ACE indices are estimators of microbial species richness. On day 0, the ACE and Chao1 values in the AO, US–chlorine + TW, and US–chlorine + AO groups were consistent with those in control group (Table 1), indicating that the treatments only reduced microbial counts and did not kill any microbes to an undetectable level. After storage for 7 days, a significant decrease in ACE and Chao1 values was detected in the control group, which was consistent with the findings of Lopez-Velasco et al. [56], who reported that refrigeration reduced the number of microbial species in spinach. On day 7, the Chao1 and ACE values in the treatment groups were significantly lower than those in the control group, and no significant differences were observed between the three treatment groups, which was consistent with our previous findings [57].

**Table 1:** Microbial diversity on the lettuce leaves after treatment.

Parameter		Treatment			
		Control	1.0 ppm of AO (120 s)	US–chlorine (60 s) + TW (60 s)	US–chlorine (40 s) + 1.0 ppm of AO (60 s)
Chao1	d0	566.96 ± 46.85 Aa	570.17 ± 41.19 Aa	569.73 ± 72.86 Aa	573.89 ± 60.69 Aa
	d7	450.09 ± 22.38 Ba	391.57 ± 44.38 Bb	375.16 ± 27.34 Bb	404.19 ± 33.67 Bb
ACE	d0	598.26 ± 44.19 Aa	611.02 ± 45.58 Aa	620.05 ± 89.44 Aa	616.12 ± 66.09 Aa
	d7	476.71 ± 29.91 Ba	411.83 ± 48.37 Bb	396.39 ± 28.37 Bb	426.24 ± 38.79 Bb

Different lowercase letters in the same row indicate significant differences (*P* < 0.05). For the same indicator, different capital letters in the same column indicate significant differences (*P* < 0.05) between day 0 and 7. US, ultrasonication; TW, tap water; AO: aqueous ozone.

Microbial composition analysis showed that the predominant microbial species on the lettuce surface were *Pseudomonas*, *Alkanindiges*, *Massilia*, *Sphingomonas*, *Acinetobacter*, and *Escherichia–Shigella* (Table 2). On day 0, the disinfection treatments did not significantly alter the relative abundances (RAs) of these microbes. After 7-day storage, in the control group, the RA of *Pseudomonas* remained unchanged, the RA of *Massilia* decreased from 5.08 % to 0.92 %, that of *Sphingomonas* from 4.92 % to 0.34 %, and that of *Acinetobacter* from 6.20 % to 0.17 %. In contrast, the RA of *Escherichia–Shigella* increased from 2.02 % to 38.76 %, which indicates that *Escherichia–Shigella* outcompete other microorganisms for nutrients required for growth [57]. *Massilia* and *Acinetobacter* have inhibitory effects on plant fungi [[58], [59]]. We observed the highest RAs of *Massilia* and *Acinetobacter* on day 7 after US–chlorine + AO treatment, and the values were significantly higher than those in the control and other two treatment groups. This may explain why M&Y counts were the lowest in the US–chlorine + AO group during storage.

**Table 2:** Relative abundances (at the genus level) of the predominant taxa.

Genus		Treatment			
		Control	1.0 ppm of AO (120 s)	US–chlorine (60 s) + TW (60 s)	US–chlorine (40 s) + 1.0 ppm of AO (60 s)
*Pseudomonas*	d0	42.42 ± 3.95 Aa	41.05 ± 6.20 Aa	43.07 ± 7.34 Aa	47.10 ± 3.94 Aa
	d7	42.05 ± 5.12 Aa	37.54 ± 5.63 Aa	37.98 ± 2.40 Aa	42.84 ± 3.17 Aa
*Alkanindiges*	d0	7.43 ± 2.46 Aa	6.03 ± 2.42 Aa	7.28 ± 3.30 Aa	5.15 ± 1.96 Aa
	d7	5.91 ± 3.03 Aa	1.26 ± 0.68 Bb	1.92 ± 0.45 Bb	1.43 ± 1.04 Bb
*Massilia*	d0	5.08 ± 1.18 Aa	5.80 ± 1.98 Aa	6.13 ± 2.38 Aa	7.20 ± 2.54 Aa
	d7	0.92 ± 0.65 Ba	0.13 ± 0.05 Ba	1.83 ± 0.85 Bb	3.34 ± 0.95 Bc
*Sphingomonas*	d0	4.92 ± 1.45 Aa	5.86 ± 2.27 Aa	7.14 ± 2.61 Aa	6.61 ± 2.85 Aa
	d7	0.34 ± 0.24 Ba	0.12 ± 0.03 Ba	2.31 ± 0.63 Bb	2.53 ± 0.64 Bb
*Acinetobacter*	d0	6.20 ± 2.11 Aa	8.44 ± 2.70 Aa	6.76 ± 3.01 Aa	7.73 ± 2.46 Aa
	d7	0.17 ± 0.10 Ba	0.10 ± 0.04 Ba	1.02 ± 0.84 Bb	3.07 ± 0.73 Bc
*Escherichia–Shigella*	d0	2.02 ± 0.95 Aa	2.18 ± 1.51 Aa	2.45 ± 2.10 Aa	1.14 ± 1.23 Aa
	d7	38.76 ± 4.55 Ba	50.58 ± 3.66 Bb	48.09 ± 5.19 Bb	41.94 ± 1.90 Ba

Different lowercase letters in the same row indicate significant differences (*P* < 0.05). For the same indicator, different capital letters in the same column indicate significant differences (*P* < 0.05) between day 0 and 7. US, ultrasonication; TW, tap water; AO: aqueous ozone.

On day 7, the RAs of *Escherichia–Shigella* in the AO and US–chlorine + TW groups were 50.58 % and 48.09 %, respectively, which were significantly higher than that in control group, indicating that these two treatments alter the microbial composition and create a microenvironment promoting *Escherichia–Shigella* proliferation, with less microbial–microbial interaction. Similarly, Wang et al. [20] found 47.53 % *Xanthomonas* on lettuce surface after propionic acid treatment, which was significantly higher than the proportion in the control group (24.73 %). Samara et al. [60] found that the count of *L. monocytogenes* after disinfection with 0.5 % citric, propionic, and acetic acid was higher than that in the control group during storage; however, *L. monocytogenes* was significantly controlled when the disinfectant concentration was increased to 1 %. The authors concluded that 0.5 % organic acids destroys the balance between naturally present microbes and *L. monocytogenes*, leading the proliferation of *L. monocytogenes*. One study revealed that although AMC can be significantly reduced by disinfection, AMC counts in the disinfection group exceeded those in the control group during subsequent storage [8]. In this study, the RA of *Escherichia–Shigella* after US–chlorine + AO treatment was 41.94 % on day 7, which was in line with that in the control group and significantly lower than that in the other two treatment groups. This result indicated that US–chlorine + AO treatment not only has the best disinfection effect, but also maintains a more balanced microbial composition than the AO and US–chlorine + TW treatments.

The microbes present on produce surface have different cell membrane structures, rendering differential resistance to disinfectants. According to the cell membrane and count analyses (Fig. 6, Fig. 7, Fig. 8), we have demonstrated that US–chlorine + 1.0 ppm AO causes the most severe cell membrane damage and the lowest counts of *E. coli* O157:H7 and *S.* Typhimurium (both pathogens belong to *Escherichia–Shigella*). Thus, we speculate that US–chlorine + AO treatment destroys the cell membranes of most naturally present microbes, without causing an imbalance in the microbial composition, making *Escherichia–Shigella* suffer from microbial–microbial interaction during storage. However, *Escherichia–Shigella* are more resistant to AO and US–chlorine + TW treatments than other microbial species, resulting in *Escherichia–Shigella* suffering less microbial–microbial interaction and thus, with a rapid growth rate.

## 4 Conclusions

This study explored whether the use of AO rinsing can improve the disinfection efficacy of US–chlorine. We found that US–chlorine + 1 ppm AO did not lead additional quality loss, and showed an inhibitory effect on browning-related enzymes (POD and PPO). Moreover, US–chlorine + 1 ppm AO showed good disinfection efficacy against *E. coli* O157:H7 and *S.* Typhimurium, owing to the severer cell membrane damage caused by the additional AO. As for naturally present microbes, US–chlorine + 1 ppm AO suppressed AMC and M&Y counts on days 0–7. 16S rRNA analysis revealed that this was because this treatment led to the highest RAs of *Massilia* and *Acinetobacter*, which are fungi-inhibiting bacteria, and the lowest RAs of *Escherichia–Shigella*, creating a more balanced ecological environment than the other treatments. Overall, this study confirmed that AO rinsing is an efficient and quality-conserving method and can replace TW rinsing in combination with US–chlorine. One limitation of this study is that 16S rRNA sequencing only interrogates the V3-V4 region and does not allow analyzing microbial metabolic process at the molecular level. In future studies, metatranscriptomic methods can be used to further analyze alterations in microbial molecular functions and metabolic pathways after treatment with US–chlorine + AO.

### CRediT authorship contribution statement

**Yeting Sun:** Investigation, Methodology. **Zhaoxia Wu:** Data curation. **Yangyang Zhang:** Writing – review & editing. **Jiayi Wang:** Conceptualization, Supervision, Funding acquisition, Writing – original draft, Writing – review & editing.

## Declaration of Competing Interest

The authors declare that they have no known competing financial interests or personal relationships that could have appeared to influence the work reported in this paper.

## Figures and Tables

**Supplementary figure 1:**